# Hierarchical Biomaterial Scaffolds for Periodontal Tissue Engineering: Recent Progress and Current Challenges

**DOI:** 10.3390/ijms25168562

**Published:** 2024-08-06

**Authors:** Mafalda S. Santos, João C. Silva, Marta S. Carvalho

**Affiliations:** 1 Department of Bioengineering and iBB—Institute for Bioengineering and Biosciences, Instituto Superior Técnico, Universidade de Lisboa, Av. Rovisco Pais, 1049-001 Lisboa, Portugal; mafaldasantos4@tecnico.ulisboa.pt; 2 Associate Laboratory i4HB—Institute for Health and Bioeconomy, Instituto Superior Técnico, Universidade de Lisboa, Av. Rovisco Pais, 1049-001 Lisboa, Portugal

**Keywords:** biocompatible and biomimetic materials, periodontal regeneration, periodontium, hierarchical scaffolds, tissue engineering

## Abstract

The periodontium is a complex hierarchical structure composed of alveolar bone, periodontal ligament, cementum, and gingiva. Periodontitis is an inflammatory disease that damages and destroys the periodontal tissues supporting the tooth. Periodontal therapies aim to regenerate the lost tissues, yet current treatments lack the integration of multiple structural/biochemical instructive cues to induce a coordinated regeneration, which leads to limited clinical outcomes. Hierarchical biomaterial scaffolds offer the opportunity to recreate the organization and architecture of the periodontium with distinct compartments, providing structural biomimicry that facilitates periodontal regeneration. Various scaffolds have been fabricated and tested preclinically, showing positive regenerative results. This review provides an overview of the recent research on hierarchical scaffolds for periodontal tissue engineering (TE). First, the hierarchical structure of the periodontium is described, covering the limitations of the current treatments used for periodontal regeneration and presenting alternative therapeutic strategies, including scaffolds and biochemical factors. Recent research regarding hierarchical scaffolds is highlighted and discussed, in particular, the scaffold composition, fabrication methods, and results from in vitro/in vivo studies are summarized. Finally, current challenges associated with the application of hierarchical scaffolds for periodontal TE are debated and future research directions are proposed.

## 1. Introduction

The periodontium is a highly hierarchical and dynamic structure responsible for tooth support and attachment to the jawbone. It is composed of hard (alveolar bone and cementum) and soft tissues (periodontal ligament (PDL) and gingiva). These periodontal tissues surround and stabilize the teeth in their respective alveolar sockets and allow them to withstand the masticatory forces. Furthermore, the periodontium also serves as a barrier against several oral pathogens [1].

Periodontal disease is an infection that damages the periodontal tissues and first manifests as gingivitis [2]. This inflammatory disease is caused by bacteria from dental plaque accumulation and may be reversed with effective oral hygiene. However, if not treated, it can lead to periodontitis, which is characterized by alveolar bone resorption, destruction of collagen fibers from the PDL, and infiltration of soft tissue pockets between the tooth root and gingiva [3]. The destruction of the periodontal tissues is caused by host-derived mediators and enzymes produced by inflammatory cells, such as macrophages, in response to the bacterial infection of the periodontium [4]. The inability of the patient’s immune system to control the infection allows its further progression. Advanced stages of periodontitis are characterized by the destruction of periodontal tissues and loss of tooth attachment, causing loose teeth, pain and discomfort during mastication, and eventual tooth loss [5]. Severe periodontitis poses a public health problem, estimated to affect 10% of the global population and with an increasing global burden over the last three decades [6,7].

Current periodontal treatments commonly used in clinics to regenerate all the tissues damaged due to periodontitis include bone grafts, guided tissue regeneration (GTR) membranes, and several growth factors [8]. However, these treatments are still exposed to clinical failures, show limited and unpredictable results, and do not effectively promote the regeneration of a functional periodontium [9].

Tissue engineering (TE) strategies have been explored for periodontal regeneration to address the limitations of current treatments and improve the clinical outcomes of standard therapies. However, TE strategies addressing periodontal regeneration using homogeneous monophasic scaffolds have shown limited outcomes, targeting only new bone formation [10]. Recently, research has been carried out regarding the development of hierarchical biomaterial scaffolds for periodontal regeneration. Considering the complexity of the periodontium and the need to promote coordinated and organized tissue regeneration, the development of hierarchical scaffolds that emulate the various periodontal tissues is an attractive strategy for more synchronized tissue regeneration. Hierarchical scaffolds, fabricated through several techniques such as 3D printing/bioprinting [10], electrospinning [11], and hydrogel synthesis [12], present different designs and compositions, and have shown promising results in both in vitro and in vivo studies.

This review describes the periodontium’s hierarchical structure, covers the shortcomings of the currently available periodontal treatments, and summarizes alternative strategies introduced by the developments of periodontal TE. Recent research on hierarchical scaffolds for periodontal regeneration is highlighted and discussed. Finally, the limitations and future perspectives of using hierarchical scaffolds for periodontitis treatment are identified and debated.

## 2. Periodontium Hierarchical Structure

**Figure 1:**
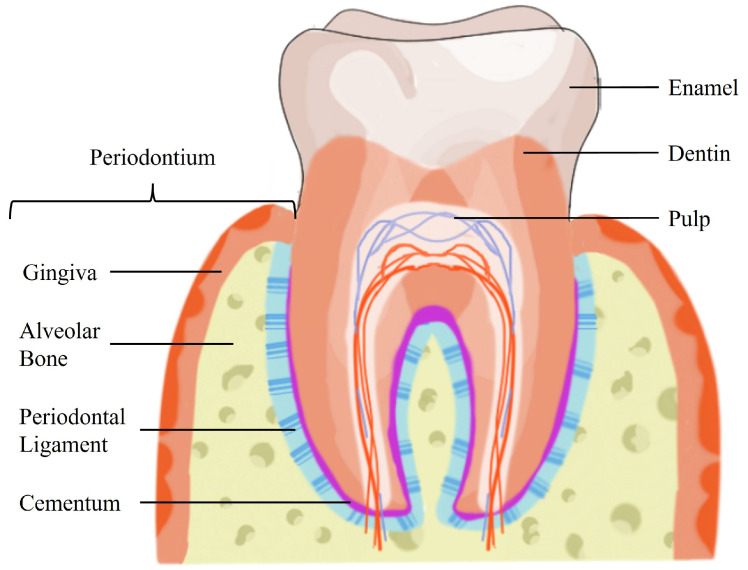
Schematic illustration of the tooth structures.

The periodontium possesses a complex hierarchical architecture, which comprises a soft tissue (PDL) interspersed between two distinct hard tissues (alveolar bone and cementum) [1]. The periodontal structures are illustrated in Figure 1.

The alveolar bone arises from the maxilla or mandible and is the part of the bone that contains the sockets where the teeth are anchored. It is a mineralized, hard tissue composed of 60% inorganic material, 25% organic material, and 15% water [13]. The alveolar bone has an important role in anchoring the roots of teeth by attaching the PDL fibers to the teeth sockets. It is connected to the root cementum through the PDL fibers, as can be observed in Figure 1. Additionally, the alveolar bone is responsible for blood vessel supply to the PDL. Since the teeth are continuously making minor movements, the alveolar bone is always remodeling to meet the functional demand placed by the forces of mastication. Bone remodeling hinges on a balance between bone resorption and bone deposition, maintained by progenitor cells that can differentiate into osteoclasts (bone resorption) and osteoblasts (bone deposition) [14]. The bone matrix is composed of hydroxyapatite (HAp), collagen, and non-collagenous proteins, such as osteopontin (OPN), osteocalcin (OC), and bone sialoprotein (BSP) [15,16].

The cementum is an avascular, connective hard tissue covering the teeth’s roots. It is located between the tooth root and the PDL, as illustrated in Figure 1. Thus, its primary function is to attach PDL fibers. Two types of cementum can be identified by the presence or absence of cells and by the origin of the collagen fibers present in the matrix. Acellular cementum provides attachment for the tooth, while cellular cementum has an adaptive role in response to tooth wear and movement and is associated with the repair of periodontal tissues [1,14]. Cementum composition is similar to bone with about 65% of its weight composed of inorganic material, 23% organic material, and 12% water [13]. The main organic component is type I collagen (COL I), constituting up to 90% of the organic matrix [1]. Interestingly, almost all non-collagenous matrix proteins identified in cementum are also found in bone, including OPN, OC, BSP, and fibronectin [16]. Periodontal regeneration requires the formation of new cementum from precursor cells, cementoblasts, which can be found in the PDL [14,16].

The PDL is an aligned fibrous connective tissue placed in the periodontal space between the alveolar bone and the root cementum (Figure 1). It spans approximately 150–400 µm between the two hard tissues [13,17]. The PDL consists of organized and well-defined collagen fiber bundles, which are mainly composed of collagen type I and confer structural strength to the PDL [13]. Its width can increase up to 50% more, along with a significant increase in the thickness of the fiber bundles, when the functional demand rises [1]. The ends of the fiber bundles are inserted into either the cementum or the alveolar bone, perpendicular to their surface [13], allowing the teeth to withstand changes in physical forces during mastication, speech, and orthodontic tooth movement [18]. The PDL is an innervated tissue, which allows proper positioning of the jaw and contributes to the sensations of touch and pressure on the teeth [13]. This complex specialized tissue comprises several heterogeneous cell populations, including osteoblasts, osteoclasts, fibroblasts, macrophages, cementoblasts, and PDL stromal cells (PDLSCs). It serves as a source for renewable progenitor cells [14], ensuring tissue homeostasis and regeneration, including the maintenance and repair of hard tissues [17]. The architecture of the PDL is crucial for the physiology of the periodontium. Functional modifications of the PDL also implicate adaptive changes in the bordering cementum and alveolar bone [1]. Hence, the PDL plays a critical role in several triggering and regulatory mechanisms modulating the regeneration of all periodontal tissues [9].

The hierarchical organization of the periodontium is attributed to the intercalated hard mineralized tissues (alveolar bone and cementum) and the soft unmineralized PDL. Damage to the intricate structure of the periodontium can result in changes to the tooth-supporting function.

## 3. Limitations of the Current Therapeutic Strategies for Periodontal Regeneration

Periodontal therapy aims to regenerate the tissues damaged or lost due to periodontitis or other conditions, such as trauma and osteoporosis [19]. Periodontitis is the main cause of tooth loss, particularly affecting elderly populations, and in its most severe form is the eleventh most prevalent condition globally [20]. Periodontal disease is a global health problem, prevalent in 20–50% of the global population and posing a significant burden to society and the economy [6,21]. Non-surgical periodontal therapy (e.g., scaling and root planing) has been shown to reduce pocket depth and result in the formation of new tooth attachment, often being sufficient for patients with early or moderate disease [2]. However, only a small amount of periodontal tissue can be regenerated at the treated sites, and outcomes from non-surgical therapy can vary according to the patient’s age and sex [22]. Hence, surgical therapy is frequently required to fully eliminate dental plaque or to restore lost periodontal structures [23]. Pocket reduction surgery involves the use of various techniques for the resection of soft and hard necrotic tissues [5].

To promote new alveolar bone formation, bone grafts from different sources (autogenous, allogeneic, xenogeneic, and alloplastic) have been used for filling debrided periodontal defects. Autogenous bone grafts are considered the gold standard for bone graft materials. These grafts are derived from the same individual, due to their high osteogenic, osteoconductivity, and osteoinductivity properties. Nevertheless, these grafts have limited availability and are associated with more significant surgical risks, including higher morbidity at the donor site [24]. Allogeneic (e.g., demineralized bone matrix) and xenogeneic grafts (e.g., deproteinized bovine bone) show osteoconductive and osteoinductive properties but may present potential complications such as immunogenic responses [25,26]. Studies have shown that allografts can cause sensitization of the patient’s immune system, whereas xenografts have been linked to complications, including foreign body reactions and maxillary fungus balls [27,28,29,30]. Rare cases of disease transmission have been reported in the use of allogeneic and xenogeneic bone grafts [31,32]. To overcome any potential immunogenicity, alloplastic bone grafts, which are synthetic products that aim to recreate the properties of natural bone, appeared as a promising alternative. However, synthetic grafts still demonstrate a limited biomechanical performance and bioactivity and support periodontal repair rather than regeneration [9,24]. Moreover, bone grafts alone fail to prevent the epithelial downgrowth into the defect [33]. In fact, bone grafting procedures have resulted in the formation of a long junction epithelium rather than a new connective tissue attachment [34].

Another current therapeutic strategy involves the use of GTR membranes, which are cell-occlusive barrier membranes that selectively exclude relatively rapid epithelial and fibroblastic downgrowth while promoting repopulation of defect sites with slower migrating cells from the PDL, bone, and cementum [35]. Non-resorbable membranes show superior space maintenance yet require a second surgery to be removed [36]. Resorbable membranes (e.g., porcine collagen membrane) eliminate the need for a second surgery but show lower mechanical strength, which can lead to membrane collapse [37]. To address the issue of epithelial downgrowth with bone grafts and to prevent GTR membranes from collapsing into the defect, GTR membranes can be combined with bone substitute materials (e.g., demineralized/deproteinized freeze-dried bovine bone allograft) [38]. GTR membranes still have limitations such as the lack of antibacterial properties, membrane exposure, or displacement, which hinder tissue regeneration [33]. Relevant problems arising from membrane exposure are the occurrence of infection and a decreased barrier function against gingival epithelial and connective tissue cells, which could impair the healing and regeneration processes in periodontal defects. Notably, the degree of membrane exposures and the possible detrimental effects on GTR healing outcomes might depend strongly on the degradation rate and structural features of the membrane material [39,40]. GTR is a predictable treatment for narrow intrabony defects and class II mandibular furcation defects, however, the results for other types of defects, such as class III furcations and cases with an extensive width of root exposure, remain limited and unpredictable [36]. However, the recent progress in the engineering of materials and modulation of the biological properties of GTR membranes through the addition of additives and biopolymers to tune their antibacterial, biocompatibility, degradation, and mechanical properties hold high potential to overcome the abovementioned limitations [41].

Recently, another periodontal treatment strategy that emerged is the use of enamel matrix derivative (EMD), which consists of a mixture of growth factors/proteins that can be applied to a periodontal defect after debridement. EMD is derived from unerupted porcine tooth buds and is composed of approximately 90% amelogenins, and also other enamel proteins [42]. It is an animal-derived product with a gel-like consistency, resulting in limited space-making potential, hence it has been recommended to be used in combination with bone grafting materials to prevent flap collapse [9]. Studies have shown the capacity of EMD to promote new alveolar bone, cementum, and periodontal ligament formation [43], yet there is significant heterogeneity in the treatment outcomes, as in the case of GTR membranes [44]. In recent years, innovative alternative biomaterials such as amnion–chorion resorbable membranes [45] and osteoinductive demineralized freeze-dried bone allograft (DFDBA) [46] have been more often used in periodontal regeneration clinical applications with promising results. Nevertheless, variability between batches of these allograft materials is still a concern and further studies are still necessary to increase the level of evidence supporting their clinical use.

The current therapeutic strategies are still exposed to poor and unpredictable clinical outcomes and do not lead to the regeneration of a functional periodontium. Bone grafts fail to promote new PDL attachment, which is essential to connect the tooth to the newly formed bone. Without a regenerated PDL, the lack of attachment to the bone will eventually lead to tooth loss [34,47]. GTR and EMD strategies can be generally considered unpredictable, taking into account the high heterogeneity of the treatment outcomes [9]. Limited clinical outcomes are linked to the inability to recreate the in vivo structural properties of the periodontium and to promote a selective repopulation of the periodontal defects by progenitor cells in a specific spatial and temporal order [47], which is crucial to achieving complete regeneration of multiple functional tissues [38].

Considering the limitations of the current therapeutic strategies, innovative treatment alternatives with more predictable regenerative outcomes are necessary. New approaches should ensure compartmentalization between the periodontal defect and the surrounding soft tissue, support a spatiotemporal coordinated recruitment of progenitor cells, and promote the regeneration of a native-like hierarchical and functional periodontium.

## 4. Periodontal Tissue Engineering

TE makes use of biomaterial scaffolds, cells, and biochemical/physical factors to facilitate tissue regeneration. These mediators are manipulated and may be combined to promote the regeneration of damaged or lost tissues. Several TE strategies for periodontal regeneration have been developed and described in the literature as promising alternatives to the current treatments.

Scaffolds for periodontal TE have been widely researched and offer alternative defect fillers to bone grafts [48]. Scaffolds can be fabricated from natural or synthetic materials and tailored to different shapes and architectures including films, fibers, sheets, gels, and sponges. Natural polymers commonly used for periodontal TE include collagen, which is the most abundant extracellular matrix (ECM) protein in the alveolar bone, PDL, and cementum; gelatin, a hydrolysis product of collagen; and chitosan (CTS), which is derived from chitin and possesses advantageous antibacterial properties [36]. These natural polymers are derived from natural sources, for example, chitosan is obtained from the exoskeletons of crustaceans, and collagen can be of bovine, porcine, or fish origin. These materials often exhibit similarities to ECM components and have high bioactivity, biocompatibility, and biodegradability. However, natural polymers might present a high batch-to-batch variability and the mechanical strength and stability of natural materials are not as high as those of synthetic polymers [49]. Synthetic polymers frequently employed as periodontal scaffold materials are polycaprolactone (PCL), polylactic acid (PLA), and polylactic-co-glycolic acid (PLGA) [33]. These polyester-based polymers provide controllable and reproducible structural properties allowing mass production. In addition, synthetic polymers present appropriate mechanical properties, and their mechanical strength and degradation rate can be adjusted in order to reach the most favorable outcome. Nevertheless, these materials lack cell attachment sites and often require chemical alterations to improve their bioactivity [33]. Another class of biomaterials used in periodontal scaffold fabrication is bioceramics, such as HAp [50,51,52] and tricalcium phosphate (TCP) [53,54], which have an advantageous composition similar to the inorganic phase of bone tissue as well as a high bioactivity. Nevertheless, the low tensile strength and brittleness of these bioceramics can limit their use for certain periodontal TE applications [55].

Biochemical cues, for example, growth factors and proteins, can be incorporated within the scaffold’s structure to promote their controlled delivery in the periodontal defect. ECM proteins and growth factors have been employed in TE, since they may enhance cell differentiation and function [32]. To induce osteogenic differentiation and promote bone formation, TE strategies have included ECM proteins found in bone, such as bone morphogenetic proteins (BMP-2 [56,57] and BMP-7 [58]). Since these proteins are also found in the cementum matrix, studies have investigated their effects on cementum regeneration. Zang et al. developed a CTS hydrogel loaded with BMP-7 and the antibiotic ornidazole and demonstrated the enhancement of new alveolar bone and cementum formation due to the presence of BMP-7 [58]. TE strategies specially focused on cementum regeneration have incorporated cementum protein 1 (CMP1) within scaffolds. Chen and colleagues produced PCL/COL I electrospun scaffolds with polyethylene glycol (PEG) and calcium phosphate nanoparticles (NPs) containing recombinant human CMP1 (rhCMP1). These scaffolds resulted in increased expression of CMP1 and cementum attachment protein (CAP) and decreased expression of OC and OPN by PDLSCs in vitro. Moreover, these scaffolds promoted the formation of cementum-like tissue, instead of new bone formation in a rat calvaria defect [59]. During the initial stages of wound healing, soluble factors are released, such as transforming growth factor-β1 (TGF-β1), platelet-derived growth factors (PDGF), and fibroblast growth factors (FGF). FGF-2 has been employed in gels and electrospun scaffolds, resulting in the formation of more regular PDL-like tissues and denser fibers bound to the alveolar bone in beagle dog models [60,61]. Moreover, rhPDGF-BB with a resorbable β-TCP particle carrier is currently FDA-approved for application in periodontal defects [62]. The delivery of soluble factors faces challenges, such as the high cost and significant side effects associated with supraphysiologic doses [32]. A more cost-effective alternative to recombinant growth factors is the use of platelet concentrates, such as platelet-rich plasma (PRP) and concentrated growth factor (CGF), which contain the above-mentioned soluble factors TGF-β1 and PDGF [32]. Ammar et al. loaded CTS hydrogels with freeze-dried platelet concentrate, which exhibited a sustained release of TGF-β1 and PDGF over 2 weeks as well as a significantly increased PDLSC viability [63].

The regeneration of periodontal tissues involves distinct cell types: osteoblasts, cementoblasts, PDLSCs, and epithelial cells [64]. One stem-cell-based TE approach is the application of cell sheets to the periodontal defect [65]. Cell sheets are obtained by harvesting confluent cultured cells with an intact ECM, resulting in minimized cell loss and damage to cell function. However, this technique is costly, requires a longer culture period and the cell sheets attach weakly to hard tissues [23,65]. These shortcomings may be addressed with the use of biomaterial scaffolds, which can be combined with a single cell sheet or multiple layers. Periodontal TE scaffolds can also be loaded with cells, for example with PDLSCs, which were shown to improve the alveolar bone regeneration capacity of gelatin methacrylate (GelMA) hydrogels in rat alveolar bone defects [66,67].

**Figure 2:**
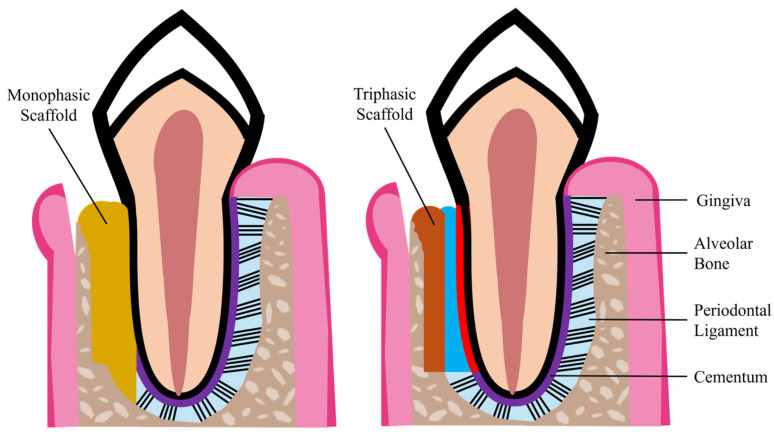
Schematic illustrations of the application of a monophasic (**left image**) and a triphasic scaffold (**right image**) in periodontal defects (adapted from [11]).

The complex hierarchical structure of the periodontium, the heterogeneous cell populations, and the highly orchestrated interaction between cells, biochemical factors, and ECM pose challenges in achieving complete periodontal regeneration. The regeneration process faces several hurdles, such as the clinically challenging surgical environment, the spatiotemporal healing coordination, and the competition between soft and hard tissues [47]. TE approaches have been evolving from simple membranes, grafts, and scaffolds to more complex constructs. A synchronized and coordinated regeneration of the periodontium may not be achieved with the use of monophasic structures (e.g., hydrogels or scaffolds) since these generally only exert control over the formation of a single tissue, which is usually bone [47]. The complete reestablishment of a functional periodontium requires the regeneration of alveolar bone, cementum, gingiva, and PDL with functionally oriented collagen fibers, through strategies capable of managing soft and hard tissue reconstruction, as well as their interfaces [68]. To address the lack of an integrated and coordinated regeneration of the periodontium’s soft and hard tissues, TE scaffolds can be compartmentalized to yield multiphasic structures. Different scaffold fabrication techniques (e.g., 3D printing and bioprinting, electrospinning, and solution casting) can be combined to fabricate more complex hierarchical scaffolds that recapitulate the native tissue architecture and provide multiple instructive cues to induce an organized regeneration of the various periodontal tissues [69]. Through the use of hierarchical multiphasic scaffolds, each phase can be tuned and specialized to enhance the regeneration of a specific periodontal tissue. For example, triphasic scaffolds can be designed and fabricated to incorporate distinct phases for alveolar bone, PDL, and cementum regeneration, as illustrated in Figure 2.

## 5. Hierarchical Scaffolds for Periodontal Tissue Engineering

Considering the hierarchical architecture of the periodontium with soft-hard tissue interfaces, the use of multiphasic hierarchical scaffolds holds great promise for effective periodontal TE strategies. Hierarchical scaffolds are composed of multiple phases with differences in terms of composition and structure. The spatial compartmentalization within the scaffold should allow the formation of alveolar bone, cementum, and PDL within the respective compartments while facilitating the integration of PDL fibers into both alveolar bone and cementum interfaces [47]. These scaffolds can integrate layers/compartments with characteristics similar to the native periodontal tissues, aiming to provide functional structural biomimicry that facilitates regeneration [47]. Importantly, scaffold design should consider the spatiotemporal organization of the several cell types present in the periodontium, and support their migration, proliferation, and differentiation.

**Figure 3:**
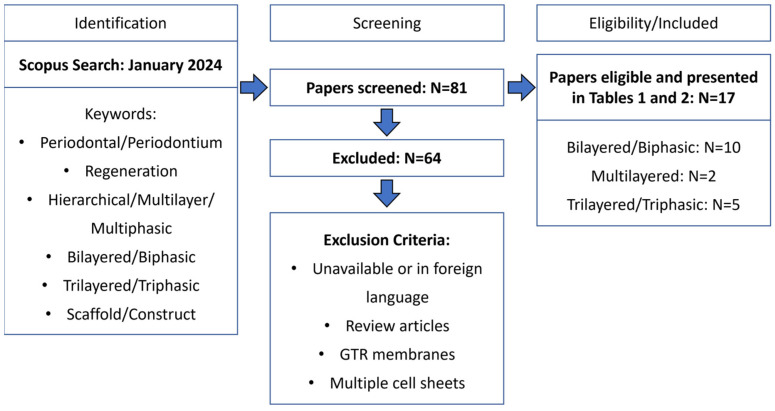
PRISMA flow diagram for the studies retrieved from the literature search and selection criteria.

The number of research studies focusing on the development of novel hierarchical scaffolds for periodontal regeneration has been increasing in the past decade. Table 1 and Table 2 provide overviews of the research studies on hierarchical scaffolds (Table 1—biphasic; Table 2—triphasic/multilayered), focusing particularly on their composition and fabrication methods, and summarizing the main results obtained from each study. The tables summarize the findings of the literature research carried out as represented in Figure 3. The following keywords were used in the search: periodontal regeneration, hierarchical, multilayered, multiphasic, scaffold, construct, and synonyms or alternative words. The studies were included if the scaffolds were designed for implantation in periodontal defects and excluded if the scaffolds were used as GTR membranes.

The majority of the biphasic scaffolds reported in the literature are designed for concurrent regeneration of alveolar bone and PDL (Table 1). Many of the proposed strategies focus on the development of a scaffold for alveolar bone regeneration and a membrane for PDL regeneration. Sundaram et al. produced a CTS hydrogel with calcium sulfate for bone regeneration, which was combined with a PCL multiscale electrospun membrane [70]. Instead of only using the synthetic polymer PCL for PDL regeneration, Liu and colleagues fabricated a heparin-conjugated PCL/gelatin blend membrane, which was placed on top of a PCL/gelatin 3D printed scaffold with HAp NPs [73]. The inclusion of HAp NPs in the scaffold resulted in statistically significant increases in *COL I*, *BMP-2*, and Runt-related transcription factor 2 (*RUNX2*) gene expression by rat bone marrow mesenchymal stem/stromal cells (rBMSCs). PCL/gelatin blend 3D printed scaffolds without HAp NPs also showed increased *COL I* and *BMP-2* gene expression compared to pure PCL scaffolds. In vivo studies demonstrated that defects treated with the biphasic construct presented higher new bone formation in comparison to defects treated only with PCL/gelatin 3D printed scaffolds [73].

Animal studies with biphasic scaffolds in dog and rat periodontal defect models demonstrated enhanced bone regeneration when compared to monophasic scaffolds [72,74]. In a study by Wei and colleagues, customized ceramic scaffolds were designed according to the periodontal defects, and a hydrogel barrier membrane was crosslinked on top of the scaffold using a specialized mold, which allowed for tight integration between the two phases of the construct [72]. This personalized strategy increased the formation of new alveolar bone and PDL, demonstrating the importance of the barrier membrane, yet lacked compartmentalization for concurrent PDL regeneration.

**Figure 4:**
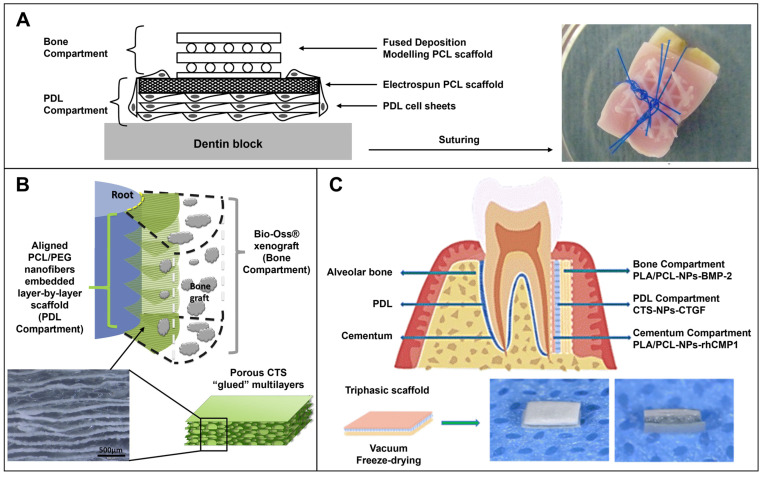
(**A**) Biphasic scaffold, composed of a 3D FDM PCL scaffold for alveolar bone regeneration and an electrospun PCL scaffold with PDL cell sheets superimposed for PDL regeneration. The biphasic scaffold was press-fitted with a dentin slice and the assembly was sutured. Reproduced from Vaquette et al. [76] with permission (Copyright 2012, Elsevier). (**B**) Multilayered CTS scaffold with aligned PCL/PEG electrospun fibers embedded for PDL regeneration. The scaffold was combined with the xenogeneic bone graft Bio-Oss^®^ for alveolar bone regeneration. Reproduced from [80] with permission (Copyright 2015, Elsevier). (**C**) The triphasic scaffold consisted of a CTS scaffold with CTGF for PDL regeneration, PLA/PCL electrospun fibers with BMP-2-loaded NPs for alveolar bone, and rhCMP1-loaded NPs for cementum regeneration. Reproduced from Hua and colleagues [86] with permission (Copyright 2023, The Royal Society of Chemistry, RSC.) BMP-2: bone morphogenetic protein; CTGF: connective tissue growth factor; CTS: chitosan; NPs: nanoparticles; PCL: polycaprolactone; PDL: periodontal ligament; PEG: polyethylene glycol; PLA: polylactic acid; rhCMP1: recombinant human CMP1.

A large number of studies produced PCL scaffolds for periodontal regeneration (Table 1 and Table 2). PCL is a biocompatible synthetic material with FDA approval that has been extensively used in biomedical applications. PCL can be easily processed and presents a slow degradation rate (2–3 years) and mechanical properties suitable for TE strategies [87]. Some studies developed PCL biphasic scaffolds, which were seeded with cells and/or combined with cell sheets [75,76,77,78,79]. For in vivo testing, biphasic scaffolds were assembled on top of a dentin slice and then subcutaneously implanted in rats [75,76,77]. Interestingly, scaffolds with cell sheets showed better attachment to the dentin slice with the oblique orientation of the PDL tissue and deposition of new cementum [76,77,78]. Vaquette and colleagues developed a biphasic scaffold with a bone compartment consisting of a fused deposition modeling (FDM)-based 3D PCL scaffold seeded with osteoblasts and a PDL compartment composed of an electrospun PCL scaffold with PDL cell sheets superimposed (Figure 4A). After press-fitting the biphasic scaffold with a dentin slide, the sutured assembly was subcutaneously implanted in rats. Scaffolds seeded with osteoblasts showed higher bone density and the superimposition of cell sheets resulted in better attachment of the scaffolds to the dentin surface [76].

The oblique orientation of newly formed PDL fibers is essential to achieve the regeneration of a functional and organized PDL. Two studies focused on the development of multilayered scaffolds solely designed for PDL regeneration. These scaffolds were implanted in rat periodontal fenestration defects and resulted in the formation of more mature collagen fibers and newly oriented PDL fibers with angulation similar to natural PDL [80,81]. Jiang et al. combined multiple layers of aligned PCL/PEG electrospun nanofibers with a CTS solution and lyophilized the construct to obtain a multilayered scaffold for PDL regeneration (Figure 4B). The multilayered scaffold was placed on the tooth root surface and the periodontal defect was filled with the xenograft Bio-Oss^®^ to immobilize the construct [80].

The application of triphasic scaffolds for periodontal regeneration was also reported in the literature (Table 2). The majority of these studies focus on the regeneration of alveolar bone, PDL, and cementum [84,85,86], yet one study designed a tricompartmental scaffold merely for alveolar bone and PDL [82] and another study considered the gingiva interface instead of the cementum [83]. Sowmya and colleagues fabricated a trilayered hydrogel scaffold with selected biochemical factors for promoting alveolar bone, PDL, and cementum regeneration. The use of specific growth factors and recombinant proteins, namely rhFGF2, rhCMP1, and PRP, resulted in similar protein expressions as obtained through fibrogenic, cementogenic, and osteogenic induction media, respectively [85]. Hua et al. also employed distinct additives in a triphasic scaffold to enhance the regeneration of periodontal tissues (Figure 4C). The compartments for the hard tissues, alveolar bone, and cementum, consisted of electrospun fiber layers with BMP-2-loaded NPs and rhCMP1-loaded NPs, respectively. In between these compartments, the PDL phase was constituted of a CTS scaffold with CTGF. The triphasic scaffold with additives, namely CTGF, rhCMP1, and BMP-2, showed improved periodontal regeneration in comparison to the control monophasic CTS blank scaffold [86]. Both strategies included rhCMP1 in their respective compartments for cementum regeneration and observed deposition of new cementum-like tissue in vivo, in addition to increased new alveolar bone and fibrous PDL formation [85,86].

The studies included in Table 1 and Table 2 show promising results in in vitro and in vivo settings. It is important to point out that the use of appropriate periodontal defect animal models for in vivo testing is necessary since the regeneration capacity of the scaffolds should be evaluated considering the formation of new alveolar bone, cementum, and PDL with oriented fibers well inserted and anchored in the regenerated hard tissues, rather than only focusing on alveolar bone formation. For example, the use of the rat calvaria bone defect model [88] and osteochondral defect model [73] only allow the assessment of bone formation, whilst press-fitting scaffolds with a dentin chip and implanting subcutaneously more closely resembles implantation in a periodontal defect, as the scaffold is in contact with dentin and PDL arrangement and cementum deposition can be detected [75,76,77,83]. Nevertheless, studies should preferably employ animal periodontal defect models (e.g., rat, rabbit, dog), as these provide more evident and reliable results on the periodontal regeneration capacity of the proposed scaffolds. Although promising results are described in Table 1 and Table 2 from in vitro studies and in vivo animal testing, the translation into clinical practice remains a difficult challenge.

## 6. Current Challenges and Future Directions

The development of hierarchical scaffolds, which are functionally graded and mimic the composition and organization of the tissues, represents progress toward simultaneous and compartmentalized regeneration of multiple tissues. Preclinical studies with animal models have demonstrated the regeneration capacity of multiphasic scaffolds, which were shown to promote the concurrent formation of new alveolar bone, PDL, and cementum.

To facilitate future clinical translation, hierarchical scaffolds should be designed and fabricated with sufficient mechanical properties to ensure ease of use in a clinical setting, strong adhesion between the different phases, and customizable morphology to adapt to periodontal defects with varying shapes and sizes [48]. The mechanical properties of the scaffolds must consider surgical handling, space maintenance for new tissue formation, and proper degradation behavior matching the rate of new tissue formation.

Difficulties in controlling the scaffolds’ degradation rate can compromise periodontal regeneration. If the implanted scaffold degrades before tissue maturation, the scaffold may collapse, hindering tissue growth [65]. Babo and colleagues developed a bilayered system composed of an injectable calcium phosphate cement with hyaluronic acid microspheres loaded with platelet lysate for bone regeneration, combined with scaffolds made of platelet lysate and genipin for PDL and cementum regeneration [89]. The bilayered system was applied in rat bilateral intrabony defects, however, its stability was compromised by the faster degradation of the injectable cement due to the incorporation of the hyaluronic acid microspheres, which allowed the infiltration of gingival tissue in the defect area, hampering the periodontal regeneration [89].

To adapt to the individual morphology of periodontal defects, scaffolds may need to be customized, which requires radiographic images of the periodontal defects, to build a 3D model for computer-aided design (CAD) and manufacturing of personalized scaffolds, for example using 3D printing [32]. However, currently, conventional 3D printing is incapable of producing high-resolution structures down to the scale of ECM structures. Improving the resolution of 3D printing methods would allow the development of biomimetic scaffolds with customizable shapes, optimized porosity, and highly precise micro/nano-structures directing desirable cell-ECM interactions [48]. Although detailed micro- and nanostructures can be fabricated, for example, using lithography [74,75], the size of multiphasic scaffolds may not fully recreate the thickness of native periodontal tissues, specifically the PDL, which spans only 150–400 µm between alveolar bone and cementum [65]. Nevertheless, the development of multiphasic scaffolds with higher complexity is associated with higher fabrication time and costs, which may limit its widespread adoption and clinical use [32]. The increased complexity of the hierarchical scaffolds might also complicate manufacturing reproducibility [47]. To address this and allow scalable and reproducible production of complex hierarchical scaffolds with or without cells, multi-technology biofabrication platforms should be investigated and implemented in the future (e.g., combining complementary manufacturing technologies such as 3D printing, bioprinting, and electrospinning) [90].

In addition to recreating the composition and architecture of the periodontium, future studies on hierarchical scaffolds should also aim to include anti-bacterial and anti-inflammatory properties, as the bacterial infection should first be controlled, and local inflammation regulated, to create a stable environment for cell proliferation and differentiation, and ultimately new tissue formation [36].

Another aspect that has been poorly investigated in hierarchical scaffold development, is mechanical stimulation. Mechanical cues were reported to play a role in the re-establishment of functional periodontal tissues [91]. Future research should integrate mechanical cues in biomaterial design and evaluate the effect of mechanical stimulation on cell differentiation and tissue formation in multiphasic scaffolds [36]. Furthermore, research should be conducted to fabricate bioreactors capable of providing multiple physical stimuli to cell-seeded scaffolds, reproducing the conditions experienced in the native environment, for example during mastication [92].

Ultimately, hierarchical scaffolds for periodontal TE should be designed to emulate the composition and architecture of the native periodontium, aiming to achieve positive clinical outcomes, by using reproducible, adaptable, and cost-effective solutions that consider the challenging and dynamic environment and structural complexities associated with periodontal regeneration [47].

## 7. Concluding Remarks

The periodontium is a hierarchical tissue with high complexity and soft-hard tissue interfaces. Current periodontitis treatments still show poor and unpredictable clinical outcomes, failing to restore functional periodontal tissues, which were damaged or lost due to the disease. Hierarchical scaffolds offer promising opportunities for improving periodontal regeneration strategies, as these can mimic the complex architecture of the periodontium. In vitro and in vivo preclinical studies on hierarchical scaffolds have demonstrated their capacity to simultaneously regenerate alveolar bone, PDL, and cementum. The development of these scaffolds still requires optimization and refinement to achieve the challenging translation from in vitro/in vivo studies into clinical practice. Future research should focus on the fabrication of hierarchical scaffolds that fully mimic the composition and organization of the natural periodontium, provide anti-bacterial and anti-inflammatory effects, and also consider the physical stimuli the periodontium is constantly exposed to, paving the way for promising novel multifunctional treatment strategies with better and more predictable clinical outcomes.

## Figures and Tables

**Table 1:** Biphasic scaffolds for alveolar bone and PDL regeneration.

Composition and Fabrication	Main Results	Year, [Ref]
CTS/calcium sulfate hydrogel prepared via NaOH neutralization (for alveolar bone regeneration). PCL electrospun membrane (for PDL regeneration).	In vitro: Osteogenic differentiation of human DFSCs on the hydrogel showed maximum ALP activity on day seven. Fibroblastic differentiation of human DFSCs on the membrane confirmed by the expression of PLAP1 and COL I proteins.	2016, [70]
Scaffold composed of Silica, CaO, and MgO, fabricated via a sol–gel technique combined with foam replica method and coated with gelatin and genipin (for alveolar bone regeneration). Bio-Gide^®^ collagen membrane (for PDL regeneration)	In vitro: Human PDLCs cultured on a sol–gel scaffold showed increased gene expression of *COL I* and *OPG* compared to cells cultured on well plates and on the Bio-Gide^®^ membrane. Cell viability and growth were maintained in both individual layers and in the bilayered construct.	2021, [71]
nCSi scaffold fabricated via digital light processing (for alveolar bone regeneration). GelMA/Si-HPMC barrier membrane produced via photo-crosslinked hydrogel injection. A barrier membrane was placed on top of the nCSi scaffold (for PDL regeneration)	In vitro: Mouse mandible-derived osteoblasts cultured with hydrogel solution showed higher migration to the scratch area, increased ALP expression, and calcium deposition. In vivo: Scaffolds were implanted in the dog’s one-wall intrabony periodontal defects. Biphasic scaffolds showed significantly more newly formed bone volume and higher trabecular number than the nCSi scaffold. Scaffolds were fully biodegraded at 8 weeks. Scaffolds paired WITH a barrier membrane resulted in a longer distance to the junctional epithelium and more newly formed PDL.	2022, [72]
PCL/gelatin scaffold with HAp NPs (PGH) fabricated via extrusion printing (for alveolar bone regeneration). PCL/gelatin electrospun membrane with heparin (for PDL regeneration).	In vitro: PGH scaffold showed enhanced proliferation of rBMSCs compared to pure PCL scaffolds. HAp NPs resulted in increased gene expressions of *RUNX2*, *COL I*, and *BMP-2* in rBMSCs. Heparin increased L929 fibroblast viability and proliferation. In vivo: Scaffolds were implanted in the rabbit osteochondral defect model. The biphasic scaffold showed more newly formed bone compared to the PGH scaffold only.	2021, [73]
Intrafibrillar mineralized collagen (IMC) scaffold fabricated via self-assembly (for alveolar bone regeneration). The CGF/collagen mixture was coated on microstamping models produced via photolithography, to obtain parallel-aligned arrays (for PDL regeneration). CGF arrays were imprinted on the IMC scaffold, and the biphasic construct was lyophilized.	In vitro: Human PDLCs cultured on IMC scaffold showed *RUNX2* and *OPN* upregulated gene expressions and decreased expression of *Elastin*. Parallel CGF arrays resulted in increased gene expressions of *POSTN* and *Elastin* and decreased expressions of *RUNX2* and *OPN*. In vivo: Scaffolds were implanted in rat periodontal defect and also subcutaneously in rats. The biphasic scaffold showed significantly more newly formed bone volume and thickness, as well as TGF-β1 and Smad3 expressions compared to the monophasic CGF scaffold and a non-hierarchical control composed of CGF and deproteinized bovine bone mineral. Subcutaneous implantation demonstrated that BMP-2^+^ and COL I^+^ cells were more abundant in the biphasic scaffold.	2022, [74]
PCL and HAp scaffold fabricated via selective laser sintering and seeded with BMP7 expressing GCs (for alveolar bone regeneration). PCL films fabricated through spin coating onto PDMS micropatterned via soft lithography and seeded with PDLCs (for PDL regeneration).	In vitro: Patterned films promoted PDLC elongation along the grooves. Nonpatterned films showed randomly oriented cells. In vivo: Scaffolds were press-fitted with a dentin chip and then subcutaneously implanted in rats. Scaffolds with micropatterned films showed increased tissue alignment, with enhanced oriented collagen fiber thickness, cell alignment, and nuclear elongation perpendicular to the dentin segment.	2016, [75]
PCL scaffold with *β*-TCP fabricated using fused deposition modeling and seeded with osteoblasts (for alveolar bone regeneration). PCL membrane produced via solution electrospinning and with 3 PDL cell sheets superimposed (for PDL regeneration).	In vitro: The bone compartment promoted the growth of osteoblasts and was highly filled with cells after 21 days. In vivo: Scaffolds were assembled on top of a dentin slice and then subcutaneously implanted in rats. Scaffolds seeded with osteoblasts showed more intense ALP staining and higher bone density. Scaffolds combined with cell sheets demonstrated better attachment to the dentin surface and deposition of cementum-like tissue.	2012, [76]
PCL scaffold with *β*-TCP fabricated using fused deposition modeling, coated with calcium phosphate, and seeded with osteoblasts (for alveolar bone regeneration). PCL membrane produced via melt electrospinning and with 3 PDL cell sheets superimposed (for PDL regeneration).	In vitro: Calcium phosphate coating significantly increased ALP activity and enhanced the mineralization of osteoblasts. In vivo: Scaffolds were assembled on top of a dentin slice and then subcutaneously implanted in rats. Calcium phosphate-coated scaffolds showed significantly more bone formation. Blood vessels penetrated the biphasic scaffold. Cell sheets facilitated attachment and oblique orientation of the tissue to the dentin block.	2014, [77]
PCL scaffold produced via melt electrospinning (for alveolar bone regeneration). PCL membrane is produced via solution electrospinning and combined with a cell sheet of PDLCs, GCs, or BMSCs (for PDL regeneration).	In vivo: Scaffolds with PDLC or BMSC cell sheets showed increased cementum coverage between weeks 5 and 10 after implantation in sheep periodontal defect. Scaffolds with GC cell sheets resulted in inferior periodontal regeneration compared to the other groups. All scaffolds promoted new cementum and bone formation, and oblique PDL fiber insertion.	2019, [78]
PCL scaffold produced via melt electrowriting, coated with calcium phosphate (for alveolar bone regeneration) and with a PDL cell sheet superimposed (for PDL regeneration).	In vitro: Calcium phosphate coating stimulated the synthesis of bone matrix by osteoblasts. Scaffolds retained functional extracellular matrix after decellularization.	2023, [79]

The abbreviations in Table 1, in alphabetical order, are as follows: ALP: alkaline phosphatase; BMP: bone morphogenetic protein; CaO: calcium oxide; CGF: concentrated growth factor; COL I: type I collagen; CTS: chitosan; DFSCs: dental follicle stem cells; GC: gingival cells; GelMA: gelatin methacrylate; HAp: hydroxyapatite; IMC: intrafibrillar mineralized collagen; MgO: magnesium oxide; NaOH: sodium hydroxide; nCSi: nonstoichiometric wollastonite; NPs: nanoparticles; OPG: osteoprotegerin; OPN: osteopontin; PCL: polycaprolactone; PDL: periodontal ligament; PDLCs: periodontal ligament cells; PDMS: polydimethylsiloxane; PLAP1: periodontal ligament associated protein 1; POSTN: periostin; rBMSCs: rat bone marrow mesenchymal stem/stromal cells; RUNX2: Runt-related transcription factor 2; Si-HPMC: silanized hydroxypropyl methylcellulose; Smad3: Mothers against decapentaplegic homolog 3; TCP: tricalcium phosphate; TGF-β1: transforming growth factor-β1.

**Table 2:** Multilayered and triphasic scaffolds for periodontal regeneration.

Composition and Fabrication	Main Results	Year, [Ref]
Multilayered CTS scaffold with embedded PCL/PEG-aligned electrospun fibers for PDL regeneration. Electrospinning and stacking of 30 layers of fibrous mats in CTS solution, followed by genipin crosslinking and lyophilization.	In vitro: Aligned fibers promoted oriented arrangement and elongation of rBMSCs, increased cell viability, and periodontal ligament-related gene expression (*POSTN* and *COL I*) compared to non-aligned fibers. In vivo: Scaffolds were implanted in rat periodontal fenestration defect followed by filling of the alveolar defect with bone graft Bio-Oss^®^. Scaffolds with aligned fibers showed higher expression of POSTN, higher collagen I/III ratio, and formation of oriented PDL-like fibers in the regenerated periodontium.	2015, [80]
Multilayered gelatin scaffold with embedded PCL-aligned electrospun fibers for PDL regeneration. Electrospinning and stacking of 20 layers of fibrous mats in gelatin solution, followed by genipin crosslinking and lyophilization.	In vitro: PDLSCs elongated along the alignment direction of the aligned PCL fibers. In vivo: Scaffolds were implanted in rat periodontal fenestration defect. Scaffolds with aligned fibers showed higher expression of POSTN and formation of newly oriented PDL fibers with angulation similar to natural PDL.	2019, [81]
Tricompartmental PCL scaffold with 50 layers produced via melt electrowriting for alveolar bone and PDL regeneration: Compartment with 10 layers, 250 µm filament spacing, and 90° layer-to-layer rotation for alveolar bone regeneration. Compartment with 30 layers, 500 µm filament spacing, and 90° layer-to-layer rotation as a transition region between bone and PDL compartments. Compartment with 10 layers, 500 µm filament spacing, and 0° layer-to-layer rotation for PDL regeneration.	In vitro: Bone compartment promoted the attachment and growth of murine pre-osteoblast cells compared to monolithic scaffold consisting of only transition region. PDL compartment facilitated the alignment of PDLCs and the orientation and formation of collagen-enriched fibers (COL I). Human calvarial osteoblasts and PDLCs were seeded in the respective compartments of the tri-compartmental scaffold, which promoted cellular and calcium distributions. The bone compartment showed increased calcium deposition compared to the PDL compartment. The transition region presented cell penetration and ligamentous insertions.	2022, [82]
Tricompartmental scaffold for alveolar bone, PDL, and gingiva interface regeneration: Medium MW CTS scaffold produced via genipin-induced gelation and seeded with osteoblasts for alveolar bone regeneration. Low MW CTS scaffold produced via genipin-induced gelation and seeded with GCs for the gingival interface. Medium MW CTS micro-channeled scaffold produced via electrochemical deposition and seeded with PDLCs for PDL regeneration.	In vitro: Cells seeded in their respective compartment showed similar viability and ALP activity compared to the control polystyrene surfaces. In vivo: Scaffolds were assembled on top of a dentin slice and then subcutaneously implanted in rats. Scaffolds showed high biocompatibility, tissue ingrowth, and vascularization. Cell-laden scaffolds resulted in the formation of a thin layer of mineralized tissue at the dentin interface.	2018, [83]
Tricompartmental PCL scaffold with 14 layers fabricated via melt extrusion for alveolar bone, PDL, and cementum regeneration: Compartment with 6 layers and high Sr-doped nano HAp (Sr-nHAp) content (20% *w*/*w*) for alveolar bone regeneration. Compartment with 3 layers, 20% *w*/*w* Sr-nHAp, and reduced strand distance for PDL regeneration. Compartment with 5 layers and low Sr-doped HAp content (10% *w*/*w*) for cementum regeneration.	In vitro: Scaffolds sustained the proliferation of osteosarcoma U2OS cells. Scaffolds with and without Sr-nHAp content showed increased ALP activity up to day 21 and decreased thereafter. Scaffolds with Sr-nHAp content showed greater mineralization than PCL scaffolds.	2020, [84]
Trilayered scaffold for alveolar bone, PDL, and cementum regeneration: Chitin/PLGA hydrogel with nBGC and PRP for alveolar bone regeneration. Chitin/PLGA hydrogel with rhFGF2 for PDL regeneration. Chitin/PLGA hydrogel with nBGC and rhCMP1 for cementum regeneration. The trilayered scaffold was assembled and lyophilized.	In vitro: Human DFSCs cultured in each hydrogel layer showed similar protein expression to hydrogels without additives in induction media. The presence of the additives CMP1, PRP, and FGF-2 resulted in improved cementogenic (CMP1, BSP), osteogenic (RUNX2, OC), and fibrogenic (FSP, PLAP1) differentiation, respectively. In vivo: Scaffolds were implanted in rabbit maxillary periodontal defects. Defects treated with trilayered scaffold containing additives showed complete closure and healing, formation of new cementum, fibrous PDL, and alveolar bone with well-defined bony trabeculae.	2017, [85]
Tricompartmental scaffold for alveolar bone, PDL, and cementum regeneration: The compartment was composed of 4 layers of PLA/PCL electrospun fibers with BMP-2-loaded CTS-BSA NPs, stacked in CTS/genipin solution (for alveolar bone regeneration). The compartment was produced using CTS/genipin solution with CTGF-loaded CTS-BSA NPs, poured on top of the bone compartment followed by crosslinking of both compartments (for PDL regeneration). The compartment was composed of 2 layers of PLA/PCL electrospun fibers with rhCMP1-loaded CTS-BSA NPs, stacked in CTS/genipin solution, followed by crosslinking and superimposition on the PDL compartment (for cementum regeneration). Triphasic scaffolds were then crosslinked and lyophilized.	In vitro: Triphasic scaffold promoted the proliferation of human PDLCs compared to porous CTS blank scaffolds. PDLCs cultured on the bone compartment showed significant upregulation of osteogenic genes (*RUNX2*, *ALP*, and *POSTN*). The PDL compartment led to a significant increase in the expression of the *Scleraxis* gene and also of *POSTN*, although less upregulated than in the bone compartment. When cultured in the cementum compartment, PDLCs showed upregulation of *CAP* and *CMP1* genes. In vivo: Scaffolds were implanted in rat periodontal defect. Triphasic scaffold with growth factors showed significantly higher newly formed bone volume and angulation of newly formed PDL fibers similar to natural PDL, in comparison to triphasic scaffold without growth factors and monophasic CTS blank scaffold. Deposition of cementum-like tissue around the root surface was observed.	2023, [86]

The abbreviations in Table 2, in alphabetical order, are as follows: ALP: alkaline phosphatase; BMP: bone morphogenetic protein; BSA: bovine serum albumin; BSP: bone sialoprotein; CAP: cementum attachment protein; CMP1: cementum protein 1; COL I: type I collagen; CTGF: connective tissue growth factor; CTS: chitosan; DFSCs: dental follicle stem cells; FGF: fibroblast growth factor; FSP: fibroblast surface protein; GCs: gingival cells; HAp: hydroxyapatite; MW: molecular weight; nBGC: nano-bioactive glass ceramic; NPs: nanoparticles; OC: osteocalcin; PCL: polycaprolactone; PDL: periodontal ligament; PDLCs: periodontal ligament cells; PDLSCs: periodontal ligament stromal cells; PEG: polyethylene glycol; PLA: polylactic acid; PLAP1: periodontal ligament associated protein 1; PLGA: polylactic-co-glycolic acid; POSTN: periostin; PRP: platelet-rich plasma; rBMSCs: rat bone marrow mesenchymal stem/stromal cells; rhCMP1: recombinant human cementum protein 1; RUNX2: runt-related transcription factor 2; Sr: strontium.

## Data Availability

Not applicable.

## Abbreviations

ALP	alkaline phosphatase
BMP	bone morphogenetic protein
BSA	bovine serum albumin
BSP	bone sialoprotein
CAD	computer-aided design
CaO	calcium oxide
CAP	cementum attachment protein
CGF	concentrated growth factor
CMP1	cementum protein 1
COL I	type I collagen
CTGF	connective tissue growth factor
CTS	chitosan
DFSCs	dental follicle stem cells
ECM	extracellular matrix
EMD	enamel matrix derivative
FDM	fused deposition modeling
FGF	fibroblast growth factor
FSP	fibroblast surface protein
GCs	gingival cells
GelMA	gelatin methacrylate
GTR	guided tissue regeneration
HAp	hydroxyapatite
IMC	intrafibrillar mineralized collagen
MgO	magnesium oxide
MW	molecular weight
NaOH	sodium hydroxide
nBGC	nano-bioactive glass ceramic
nCSi	nonstoichiometric wollastonite
NPs	nanoparticles
OC	osteocalcin
OPG	osteoprotegerin
OPN	osteopontin
PCL	polycaprolactone
PDGF	platelet-derived growth factors
PDL	periodontal ligament
PDLCs	periodontal ligament cells
PDLSCs	periodontal ligament stromal cells
PDMS	polydimethylsiloxane
PEG	polyethylene glycol
PLA	polylactic acid
PLAP1	periodontal ligament associated protein 1
PLGA	polylactic-co-glycolic acid
POSTN	periostin
PRP	platelet-rich plasma
rBMSCs	rat bone marrow mesenchymal stem/stromal cells
rhCMP1	recombinant human cementum protein 1
RUNX2	Runt-related transcription factor 2
Si-HPMC	silanized hydroxypropyl methylcellulose
Smad3	Mothers against decapentaplegic homolog 3
Sr	strontium
TCP	tricalcium phosphate
TE	tissue engineering
TGF-β1	transforming growth factor-β1
